# Systemic Risks of Topical Anesthetics in Barrier-Compromised Dermatologic Patients

**DOI:** 10.7759/cureus.84157

**Published:** 2025-05-15

**Authors:** George Chamoun, Alyssa Forsyth, Sarah Kazemeini, Justin Ma, Kelly Lam, Nasim Kasiri, Kelly M Frasier

**Affiliations:** 1 Texas College of Osteopathic Medicine, University of North Texas Health Science Center, Fort Worth, USA; 2 Kirk Kerkorian School of Medicine, University of Nevada, Las Vegas, Las Vegas, USA; 3 College of Osteopathic Medicine, California Health Sciences University, Clovis, USA; 4 College of Osteopathic Medicine of the Pacific, Western University of Health Sciences, Pomona, USA; 5 Stritch School of Medicine, Loyola University Chicago, Chicago, USA; 6 Department of Dermatology, Northwell Health, New Hyde Park, USA

**Keywords:** anesthetic pharmacokinetics, cutaneous analgesia, local pain relief, numbing agents, pain management, skin barrier dysfunction, skin barrier permeability, topical anesthetic, topical anesthetic systemic toxicity, topical lidocaine

## Abstract

Topical anesthetics are widely employed in dermatology for cosmetic interventions, laser therapies, and minor surgical procedures. Although generally safe, their systemic absorption is highly influenced by the integrity of the epidermal barrier. Inflammatory skin disorders such as psoriasis and atopic dermatitis compromise the stratum corneum (SC), facilitating enhanced percutaneous absorption and elevating the risk of systemic toxicity. This concern is particularly pertinent for potent agents such as lidocaine, tetracaine, and prilocaine, which, at elevated plasma concentrations, can induce central nervous system and cardiovascular complications, including seizures and arrhythmias. Barrier disruption promotes the passive diffusion of these lipophilic compounds, a process exacerbated by altered tight junctions, increased transepidermal water loss (TEWL), and heightened vascular permeability. Furthermore, inflammation-driven modifications in enzymatic activity may prolong anesthetic half-life, further increasing systemic exposure. Additional risk factors include the use of occlusive dressing techniques, prolonged application duration, and the concurrent use of multiple anesthetic agents. This review examines the pathophysiology of topical anesthetic absorption, the mechanisms underlying enhanced systemic exposure in the context of impaired epidermal integrity, and clinical strategies to mitigate toxicity. A nuanced understanding of these dynamics is crucial for optimizing the safe use of topical anesthetics, particularly in dermatologic and procedural care contexts.

## Introduction and background

Topical anesthetics are essential for pain relief and minimizing discomfort during various dermatologic procedures, including skin biopsies, laser treatments, and cosmetic procedures. The most common anesthetics, such as lidocaine, prilocaine, and benzocaine, are well known for their efficacy and ease of application. They primarily focus on reversibly blocking nerve conduction, resulting in temporary loss of sensation by targeting free nerve endings localized to the administration site [1]. The pharmacological mechanism and convenience enable analgesic agents to be applied gently to sensitive areas, thereby avoiding tissue trauma and swelling. Additionally, widely used anesthetic agents come in multiple forms, including sprays, solutions, gels, and ointments [2]. With the formulations previously mentioned, topical agents can avoid the risk of needlestick injuries and reduce the risk of infection. These factors are ideal for minor procedures because there is no need for special equipment, and for small lesions, topical anesthetic application is more practical than administering multiple injections. However, there are implicated risks with using topical analgesics that have the potential to cause systemic effects in patients with compromised skin barriers.

The skin barrier acts as a first-line defense against environmental elements, pathogens, allergens, and irritants. It also serves as a wall that maintains water content and balance, reduces the effects of ultraviolet light exposure, and mitigates oxidative stress [3]. This is important because the epidermis, being the outermost layer, the stratum corneum (SC), becomes compromised when it undergoes inflammation, excessive dryness, or structural damage. This report focuses specifically on compromised skin barriers due to inflammatory skin conditions, such as eczema, psoriasis, and other forms of dermatitis. In nearly all epidermal dysfunctions, including psoriasis, eczema, and other dermatitides, the skin barrier's permeability is significantly altered [4]. With a change in permeability and an overall impaired skin barrier, properly regulating the absorption of products and protection against irritants becomes an issue. As a result, the altered anatomy of the skin increases skin sensitivity and makes it more susceptible to irritation from external agents. This also significantly impacts wound healing, making treatment even more challenging. Although human skin is an exceptional protector of the body's internal systems, impairment of its barrier function introduces substantial clinical challenges that require careful management.

Disrupted skin barriers variably affect the safety profile of topical anesthetics based on permeability and absorption rates. When skin is compromised, the protective barrier limiting drug absorption is weakened, allowing for increased penetration of anesthetics. The SC functions as a hydrophobic barrier regulating the absorption of exogenous substances. The disruption reduces the barrier's effectiveness, allowing for potential systemic toxicity from topical anesthetics. In a case study and literature review by Hoffman et al., published in Dermatology and Therapy, they discuss the systemic toxicity of an anesthetic cream, eutectic mixture of local anesthetics (EMLA), to a weakened skin barrier, which can lead to central nervous system and cardiovascular complications, including lethargy, cyanosis, and cardiopulmonary instability [5]. The ramifications of these problems can have a detrimental effect on procedures and treatment; however, it is also crucial to consider the extent of skin damage. Whether it is a minor disruption, such as mild atopic dermatitis, or a severe wound, the change in skin permeability depends on the extent of the injury sustained. Based on these findings, understanding the systemic effects of anesthetic agents for treatments or procedures with a compromised skin barrier is important for preventing complications and adverse outcomes.

Concerning modern clinical guidelines and patient safety in dermatology, this study aims to assess the risk of topical anesthetics in patients with compromised skin barriers by evaluating their pharmacokinetics, systemic absorption, and potential complications. By focusing on inflammatory dermatoses that impair the skin barrier, this study aims to enhance the clinical outcomes of anesthetic practice in patients with skin barrier dysfunction. Understanding how compromised skin affects the absorption of anesthetics and how it impacts the risk of toxicity is crucial to providing optimal patient care. The findings will be utilized to provide insight into preventing the complications associated with disrupted skin barriers and improve clinical guidelines for patient safety in dermatology. By assessing systemic risk and drug permeability, this research aims to establish safer protocols for anesthetic administration in patients with impaired skin barriers. This study seeks to improve dermatologic treatment standards through a comprehensive analysis by revealing the associated risks of topical anesthetics and compromised skin barriers. Through this focus, the study contributes to improving dermatologic treatment standards for patients with inflammatory skin conditions.

## Review

Topical anesthetics in dermatology

Topical anesthetics commonly utilized in dermatologic practice include lidocaine, prilocaine, and tetracaine, each available in various formulations designed to optimize cutaneous absorption and anesthetic efficacy. A widely used preparation is a lidocaine-prilocaine eutectic mixture, consisting of 2.5% lidocaine and 2.5% prilocaine in a cream base, commercially known as eutectic mixture of local anesthetics (EMLA) [6,7]. Additional formulations include liposomal-encapsulated lidocaine, a tetracaine gel containing 40 mg of tetracaine per gram of gel, and a self-heating lidocaine-tetracaine patch that delivers 70 mg of lidocaine and 70 mg of tetracaine per patch [6]. These preparations are applied to achieve localized cutaneous anesthesia, primarily to mitigate pain associated with procedures such as needle punctures, interventions involving the eyes, ears, and nasal mucosa, and minor superficial skin surgeries.

Lidocaine and prilocaine are classified as amide-type local anesthetics, primarily metabolized in the liver by cytochrome P450 enzymes. In contrast, tetracaine and benzocaine are ester-type anesthetics, hydrolyzed rapidly in plasma by nonspecific esterases such as butyrylcholinesterase [1,2]. These metabolic pathways have important clinical implications in topical application, particularly when the drug is applied over inflamed, abraded, or otherwise compromised skin. Due to their slower hepatic metabolism, amide anesthetics may accumulate systemically in patients with impaired liver function or when applied over large areas or under occlusion conditions that increase percutaneous absorption [3,5]. Conversely, ester anesthetics such as benzocaine and tetracaine are more prone to causing hypersensitivity reactions, as their metabolism generates para-aminobenzoic acid (PABA), a known allergen [2]. Additionally, benzocaine is uniquely associated with methemoglobinemia, a potentially life-threatening condition in which hemoglobin is oxidized to a form that cannot bind oxygen. This adverse effect is most commonly observed with high concentrations, mucosal application, or prolonged use and carries particular risk in infants or individuals with glucose-6-phosphate dehydrogenase (G6PD) deficiency [1,6]. Therefore, understanding the pharmacological distinctions between these agents is essential when selecting a topical anesthetic, especially in patients with predisposing conditions or when use is expected to exceed routine exposure.

The mechanism of action of topical anesthetics involves the reversible inhibition of voltage-gated sodium channels within cutaneous nerve fibers, thereby blocking nerve depolarization and subsequent transmission of pain signals [8-10]. Targeted nerve fibers reside within the epidermis and dermis and are shielded by the stratum corneum, a highly lipophilic, water-impermeable barrier. Effective anesthetic penetration occurs primarily through passive diffusion, facilitated by the physicochemical properties of the topical formulations [11].

Lidocaine demonstrates a rapid onset of action, typically within 3-5 minutes following topical administration [8]. Its absorption is influenced by concentration, formulation (e.g., cream, gel, and patch), surface area, and integrity of the skin barrier. Lidocaine exhibits approximately 65% protein binding, primarily to albumin and α1-acid glycoprotein, which contributes to its intermediate duration of action, approximately four hours [8]. Metabolism occurs predominantly in the liver through the cytochrome P450 system, specifically via the CYP1A2 and CYP3A4 enzymes. Lidocaine and its metabolites are subsequently excreted in the urine [8].

Tetracaine has a longer duration of action, lasting approximately 4-6 hours. Following topical application, it undergoes hydrolysis by plasma cholinesterase (butyrylcholinesterase), resulting in the production of para-aminobenzoic acid (PABA) and an alcohol derivative [12]. This enzymatic degradation limits systemic exposure and toxicity [12]. Tetracaine and its metabolites are primarily eliminated via renal excretion.

Prilocaine exhibits a very rapid onset of action, within 2-4 minutes, with a relatively shorter duration of anesthesia, lasting approximately 1-1.5 hours in soft tissues [13]. It undergoes metabolism in both the liver and kidneys, producing metabolites such as ortho-toluidine, which carries a risk of inducing methemoglobinemia, particularly in vulnerable populations, including infants and those with genetic disorders [13,14]. Prilocaine clearance is predominantly renal, and hepatic or renal dysfunction may significantly alter its pharmacokinetic profile [13].

Topical anesthetics carry a potential risk for systemic toxicity, particularly when applied over large surface areas, used in high concentrations, or left in place for extended periods. Reported systemic adverse effects include cardiotoxicity, sympathetic stimulation, and psychomotor reactions such as hyperventilation, extremity paresthesia, dizziness, circumoral numbness or tingling, anxiety, tremulousness, excitement, and, in severe cases, convulsions [15]. Furthermore, select agents, specifically lidocaine, prilocaine, and benzocaine, have been associated with the development of methemoglobinemia, a condition more commonly observed in infants younger than 12 months of age due to their decreased enzymatic capacity to reduce methemoglobin [14].

In addition to systemic effects, cutaneous adverse reactions are an important consideration. Risk factors include the development of allergic contact dermatitis and delayed localized swelling at the site of application, typically emerging several hours post-application and peaking at approximately 72 hours [16]. Although rare, immediate hypersensitivity reactions such as urticaria at the application site or systemic anaphylaxis have been reported, usually manifesting within minutes to hours after exposure [17]. It is also crucial to avoid the application of topical anesthetics in patients with a history of blistering skin disorders or localized eczematous reactions following previous anesthetic use, as these individuals may be at elevated risk for severe cutaneous adverse events [18].

Table 1 summarizes the pharmacokinetic properties of lidocaine, prilocaine, and tetracaine.

**Table 1:** Pharmacokinetic Properties of Common Topical Anesthetics Used in Dermatology Data adapted from Kumar et al. [1], Lee [2], Tulga and Mutlu [6], Beecham et al. [8], Butterworth and Strichartz [9], and Stringer et al. [12]. No direct reproduction. PABA: para-aminobenzoic acid

Parameter	Lidocaine	Prilocaine	Tetracaine
Onset of action	Begins typically in 3-5 minutes [1,2,8]	Usually 2-4 minutes [1,2,6]	Onset tends to be slower than lidocaine or prilocaine [2,6]
Duration of action	Lasts about 4 hours [1,2,8]	1-1.5 hours for soft tissue anesthesia [1,6]	Duration can extend 4-6 hours [2,6,12]
Primary metabolism	Mainly hepatic (via CYP1A2, CYP3A4) [1,8,9]	Metabolized in the liver and kidneys [1,2]	Hydrolyzed by plasma cholinesterase [1,2]
Protein binding	Approximately 65% to albumin and α₁-acid glycoprotein [8]	Moderate protein binding [2]	High protein binding [1,2]
Primary excretion	Excreted primarily in urine [8]	Eliminated through the kidneys [2]	Renal excretion [2]
Key metabolites	Monoethylglycinexylidide, glycinexylidide [8]	Ortho-toluidine (linked to methemoglobinemia) [1,6]	PABA, alcohol derivatives [1,2,9]
Special considerations	Risk of systemic effects with high doses [1,8]	May cause methemoglobinemia, especially in infants [1,2,6]	Allergic reactions possible due to PABA derivatives [1,2,9]

Compromised skin barriers: role, disruption, and disease-specific changes

The skin barrier serves as the body's primary defense against the external environment, orchestrating a range of physiological processes essential for maintaining homeostasis. One of its primary functions is regulating transepidermal water loss (TEWL), thereby limiting the body's exposure to environmental threats. The stratum corneum (SC), the outermost layer of the epidermis, is enriched with hydrophobic lipids that act to prevent water evaporation and preserve skin hydration [19]. Maintaining the hydration of the SC is crucial for preserving the structural and protective integrity of the skin barrier, as many of its essential functions depend on hydrolytic reactions [20]. Adequate hydration enables the SC to retain its elasticity, tensile strength, and optimal pH, all of which contribute to its mechanical and biochemical resilience [20].

The skin barrier also incorporates a variety of structural proteins and bioactive molecules that mediate additional protective mechanisms, including antimicrobial activity, antioxidant defense, and immune surveillance [21]. Given the central role of the skin barrier in maintaining overall skin health, preservation of these physiological functions is paramount. Disruption of the barrier can compromise these defenses, resulting in increased vulnerability to environmental insults, pathogens, and irritants.

Inflammatory skin conditions, including psoriasis, atopic dermatitis, and contact dermatitis, disrupt the skin barrier. In psoriatic conditions, various physiological irregularities contribute to barrier dysfunction, including the markedly increased expression of keratinocyte differentiation markers, dysfunction of epidermal tight junctions, lipid dysfunction in the SC, and an enhanced inflammatory response resulting from increased keratinocyte proliferation [22]. As a result of skin barrier defects associated with psoriatic conditions, psoriasis leads to various characteristic skin manifestations. The physiological irregularities observed in psoriasis contribute to the hyperkeratosis in psoriatic lesions [22]. The most common skin changes observed in psoriasis result in plaque psoriasis. Plaque psoriasis is characterized by erythematous lesions, silvery-white scales, and well-defined borders [23]. The symptoms observed in psoriasis are further exacerbated with more significant skin barrier dysfunction, making the skin more susceptible to environmental and outside harm.

Atopic dermatitis and other eczematous conditions, including contact dermatitis, present with physiological irregularities that also contribute to skin barrier dysfunction. In atopic dermatitis conditions, mutations in genes that encode structural proteins, dysfunction in the immune response, and dysregulation of the skin microbiome lead to skin barrier defects [24]. These physiological dysfunctions contribute to the clinical manifestation of atopic dermatitis. Atopic dermatitis is characterized by erythematous, lichenified lesions, often accompanied by pruritus and dryness [25]. Contact dermatitis may present with similar characteristics to atopic dermatitis but varies in its manifestation. Physiological irregularities leading to skin barrier dysfunction in contact dermatitis are often caused by exposure to irritants and allergens. Exposure to such substances triggers innate immune signaling responses, leading to increased inflammation and compromised skin barrier permeability [26]. Impairment of the skin barrier in eczematous conditions leads to similar outcomes as those in psoriasis, increasing the risk of worsening eczema and other inflammatory skin condition symptoms and leaving the skin in a more vulnerable state.

Disrupted skin barriers and their impact on the safety of topical anesthetics

Local anesthetic systemic toxicity is a potentially life-threatening event, and it occurs when anesthetic agents reach peak plasma concentration levels. This can lead to severe cardiovascular and neurological complications, ranging from sensory changes to conduction disturbances [27]. The adverse implications associated with systemic toxicity highlight the importance of recognizing and understanding the mechanism of topical anesthetics through damaged skin. Topical anesthetics function by blocking the intracellular domain of voltage-gated Na+ channels, ultimately preventing neuronal transmission and inhibiting depolarization [28]. The depolarization blockade prevents a nerve from transmitting pain signals to the brain. While these agents are effective for pain management, their absorption depends on the integrity of the stratum corneum (SC), a lipid-based barrier composed of ceramides, free fatty acids, and cholesterol [29]. This is because anesthetics must enter the dermis to bind the voltage-gated sodium channels. When this protective barrier is disrupted, the absorption of anesthetics can be significantly enhanced.

The ability of a topical anesthetic to penetrate the SC, therefore, depends on various factors, such as the acid dissociation constant of the anesthetic of interest and the thickness and stability of the SC [30]. In normal physiological conditions, only the uncharged form of the anesthetic can diffuse through the SC. When tissue becomes inflamed, the local pH decreases, shifting the equilibrium toward the ionized form of the anesthetic [31]. This suggests that the application of anesthetics may be complicated in scenarios of infection, as lipid solubility is affected. Wounds and chronic dermatologic conditions, such as psoriasis, can further affect the SC [32]. Changes in skin permeability, whether caused by chronic disease or injury, can impact the absorption of anesthetics. As larger quantities of topical anesthetic enter through the damaged skin barrier, the risk of reaching toxic plasma concentrations increases. Hydration, temperature, and occlusive dressings can further enhance absorption by increasing drug retention time [33]. The heightened absorption associated with these conditions and scenarios raises the risk of systemic toxicity and necessitates monitoring in affected individuals.

Clinical evidence highlights the enhanced risk of systemic toxicity in patients with disrupted skin barriers. Barrier disruptions caused by injury and dermatologic conditions can make the skin more sensitive to external substances [21]. Furthermore, case studies demonstrate the significant risks of applying topical anesthetics to damaged skin. Hoffmann et al. described a case of acute systemic toxicity following the application of EMLA cream to a leg ulcer [5]. The patient experienced cardiovascular complications, supporting the concept that open wounds can facilitate systemic anesthetic absorption. This is attributed to bypassing the protective barrier function of intact skin. Similarly, a case study documented a patient who developed ketamine toxicity after applying a compounded analgesic cream to relieve pain from pyoderma gangrenosum [34]. Since pyoderma gangrenosum is characterized by epidermal compromise and inflammation, this case highlights how inflammatory skin diseases may enhance systemic anesthetic absorption. These examples illustrate the importance of exercising caution when applying topical anesthetics to skin surfaces that are not entirely intact.

Additional literature suggests that standard dermatologic procedures involving barrier disruption can also increase the risk of toxicity. Lasers are identified as a safe and effective method for enhancing the delivery of topically applied agents through the skin [35]. This is achieved by continuously ablating the epidermis. Fractional laser resurfacing, on the other hand, creates microscopic epidermal wounds that enhance transdermal drug delivery [36]. Although this is beneficial for therapeutic agents, it can lead to unintended anesthetic toxicity when not adequately monitored. Marra et al. reported a case of systemic toxicity following the application of 30% topical lidocaine in conjunction with fractional photothermolysis [37]. This case highlights the risks associated with using high concentrations of topical anesthetics when skin permeability is artificially enhanced through laser procedures. In situations where enhanced transdermal drug delivery is desired through dermatologic procedures, it is, therefore, essential to monitor for adverse effects such as systemic toxicity.

Several precautionary measures should be considered to minimize systemic risks associated with topical anesthetics in patients with skin barrier dysfunction. One method involves using vasoconstrictors, such as epinephrine, to reduce systemic absorption by prolonging local anesthetic effects and decreasing peak blood concentrations [38]. Epinephrine is the most common vasoconstrictor used in conjunction with local anesthetics, as it can be employed to reduce the likelihood of anesthetics reaching peak plasma concentration levels. The safety of epinephrine in dermatologic procedures involving the feet, digits, and hands has also been demonstrated in current literature [39]. This recent finding helps support its use in areas initially believed to be susceptible to injury or necrosis. Additionally, when epinephrine is added to topical anesthetics, it provides the additional benefit of hemostasis [40]. Epinephrine has been shown to promote a strong physiological response to vasoconstriction, thus bolstering its utility in various surgical and procedural scenarios. Using vasoconstrictors can serve as an effective strategy to reduce risks associated with elevated plasma anesthetic levels and ineffective hemostasis.

Adjustments in formulation also play a key role in reducing systemic toxicity risks. Because metabolic acidosis may enhance the cardiovascular toxicity of topical anesthetics, buffering anesthetics with sodium bicarbonate has been shown to reduce injection pain and stabilize anesthetic pH [41]. Cardiac arrest due to local anesthetic toxicity can therefore be addressed by considering the use of this compound. Additionally, pediatric and elderly patients exhibit differences in the activity and concentration of drug-metabolizing enzymes [42]. Due to these differences, these populations are particularly susceptible to adverse effects attributed to drug metabolism and clearance. In cases where immunologic reactions to lidocaine are a concern, switching to an ester-type anesthetic may provide a safer alternative. This is attributed to metabolism by plasma cholinesterase, resulting in a short plasma half-life and decreased capacity for toxicity [43]. Ester-type anesthetics are beneficial in reducing systemic exposure and hypersensitivity reactions. Low-dose formulations should also be considered for these high-risk populations, as this can help reduce the potential for toxicity. Barrier disruption can also result in greater percutaneous drug penetration, so limiting the surface area and duration of application could reduce systemic exposure [44]. These characteristics are fundamental in patients with chronic conditions, such as psoriasis. Developing evidence-based recommendations will ultimately be essential in improving patient safety while maintaining the effectiveness of dermatologic procedures.

Table 2 outlines the impact of disrupted skin barriers on the safety and absorption of topical anesthetics.

**Table 2:** Impact of Disrupted Skin Barriers on the Safety and Absorption of Topical Anesthetics Data adapted from Kumar et al. [1], Lee [2], Hoffmann et al. [5], Beecham et al. [8], Del Rosso and Levin [20], Orsmond et al. [22], Ramadon et al. [33], Kessler et al. [34], Marra et al. [37], Macfarlane et al. [27], and Becker and Reed [40]. No direct reproduction. EMLA: eutectic mixture of local anesthetics

Factor	Mechanism	Impact on topical anesthetic safety	Clinical examples/notes
Disruption of stratum corneum	Compromise of the lipid barrier enhances drug penetration	Increased systemic absorption and risk of toxicity	Seen in conditions such as psoriasis or mechanical trauma [1,2,22]
Inflammation and pH changes	Inflammatory states lower skin pH, increasing ionized anesthetic fraction	Reduced efficacy, less predictable absorption	Common in infected or inflamed lesions [1,2,20]
Open wounds and ulcerations	Exposes vasculature directly to drug	Rapid systemic uptake and possible toxicity	Case of EMLA toxicity via leg ulcer application [5]
Chronic skin diseases	Barrier disruption + chronic inflammation amplify drug absorption	Systemic exposure increases	Ketamine cream toxicity in pyoderma gangrenosum [34]
Procedures (laser resurfacing, etc.)	Skin ablation techniques open microchannels	Dramatic enhancement of percutaneous absorption	Lidocaine toxicity post-fractional photothermolysis [37]
Use of occlusive dressings	Occlusion increases hydration and prevents evaporation	Enhances drug retention and transdermal passage	Requires caution with high-concentration anesthetics [2,8,33]
Risk mitigation strategies	Strategies include vasoconstrictors, buffering, and dose reduction	Help minimize systemic toxicity	Use of epinephrine or ester anesthetics; caution in pediatrics and geriatrics [2,8,27,40]

Future directions

There is a significant need for focused studies on the pharmacokinetics of topical anesthetics in patients with disrupted skin barriers, particularly in high-risk populations such as infants, the elderly, and individuals with chronic dermatologic conditions. Current research lacks large-scale clinical trials that evaluate the safety and efficacy of topical anesthetics in these vulnerable groups. Future research should fill these gaps by assessing the absorption rates, potential toxicity, and safe application dosages for these patients. Furthermore, pharmacokinetic studies should investigate how different stages of skin barrier dysfunction, such as those seen in active inflammatory phases of psoriasis or eczema, affect anesthetic absorption, ensuring that specific dosing guidelines are available for patients with compromised skin.

There is also a pressing need to develop improved monitoring guidelines to ensure patient safety during dermatologic procedures involving topical anesthetics. Clear protocols must be established for identifying patients at high risk for systemic toxicity, ensuring timely intervention when necessary. These protocols should address factors such as skin conditions, the use of occlusive dressings, and the role of procedures such as laser treatments that intentionally disrupt the skin barrier, all of which can enhance anesthetic absorption. Developing real-time monitoring systems, such as those utilizing biomarkers or advanced imaging techniques, to track anesthetic concentrations in the bloodstream could be particularly beneficial for patients at high risk of complications.

Alternative approaches and new formulations of topical anesthetics should also be explored. Research could focus on developing anesthetics that are less likely to be absorbed systemically, such as drugs with higher molecular weight or formulations designed to limit skin penetration. Another promising avenue would be the development of delivery systems that primarily target the dermis while limiting systemic exposure. Microneedle patches, such as iontophoresis-driven fiber-based microneedle patches, have demonstrated controllable and long-lasting local delivery of lidocaine, with the potential to limit systemic exposure [45]. Moreover, formulations that enhance the skin barrier or reduce irritation in patients with compromised skin, such as those with psoriasis, eczema, or chronic wounds, could reduce the risk of adverse effects. Topical treatments incorporating skin-repairing compounds, such as ceramides or linoleic acid, have been shown to restore barrier function and alleviate symptoms in conditions such as atopic dermatitis [46].

By addressing these research gaps, we can significantly enhance patient safety and treatment outcomes, particularly for individuals with skin conditions that compromise integrity. Additionally, exploring these innovative formulations and strategies will enable clinicians to provide adequate pain relief while minimizing the risk of systemic toxicity, ultimately leading to more personalized and safer dermatologic care.

## Conclusions

Topical anesthetics play a vital role in dermatology by providing adequate analgesia for a wide range of procedures, including cosmetic interventions, minor surgeries, and laser treatments. While these agents are generally safe when used on intact skin, their pharmacokinetics and risk profiles are profoundly influenced by the integrity of the epidermal barrier. Disruption of the stratum corneum, whether due to inflammatory skin diseases such as psoriasis, eczema, and atopic dermatitis, or due to procedural interventions such as laser resurfacing, can significantly enhance percutaneous absorption, increasing the risk of systemic toxicity. Understanding the mechanisms by which skin barrier dysfunction alters drug absorption, including changes in lipid composition, tight junction integrity, pH balance, and vascular permeability, is critical for mitigating adverse outcomes. Clinical evidence, including case studies, underscores the importance of exercising caution when applying topical anesthetics to compromised skin, as elevated plasma concentrations can lead to serious neurological and cardiovascular complications.

Risk mitigation strategies, such as limiting the surface area and duration of application, utilizing vasoconstrictors such as epinephrine, adjusting formulations, and selecting lower doses for high-risk populations, are essential to enhance patient safety. Special consideration must be given to vulnerable populations, including infants, the elderly, and individuals with chronic dermatologic conditions. Continued research into the pharmacokinetics of topical anesthetics in patients with barrier dysfunction, the development of targeted drug delivery systems, and the establishment of evidence-based clinical protocols will be crucial in advancing patient care. A deeper understanding of the interplay between barrier integrity and anesthetic absorption will enable dermatologists to maximize therapeutic efficacy while minimizing the risk of systemic toxicity, ultimately fostering safer and more effective dermatologic practice.
